# Implementation Efficiency of Corporate Social Responsibility in the Construction Industry: A China Study

**DOI:** 10.3390/ijerph15092008

**Published:** 2018-09-14

**Authors:** Xuetong Wang, Wenyong Lai, Xiangnan Song, Chen Lu

**Affiliations:** School of Management, Guangzhou University, Guangzhou 510006, China; xtwang@gzhu.edu.cn (X.W.); lwy9408@163.com (W.L.); luchen539@163.com (C.L.)

**Keywords:** CSR efficiency, three-stage DEA model, environmental factor, construction company

## Abstract

Corporate social responsibility (CSR), as companies’ commitment to the sustainable development of the whole society, is an important approach for construction companies to respond to the emerging social and environmental issues. As the improvement of CSR efficiency leads to the reduction of CSR cost, CSR efficiency is becoming increasingly prominent for construction companies. In this research, the three-stage data envelopment analysis (DEA) model is adopted to analyze the CSR efficiency of Chinese construction companies in the period of 2012–2016. The findings of this research are as follows: (1) the efficiency of the Chinese construction companies in fulfilling CSR has not yet reached an optimal level; (2) the effect of institutional factors on CSR efficiency is complex and non-linear; and (3) the improvement of the CSR efficiency in the Chinese construction industry relies on both optimizing the institutional environment and enhancing the management capacity of CSR efficiency. These findings can not only provide empirical evidence for the government to formulate targeted policy-making regarding marketization to promote construction companies’ efficient commitment of CSR, but also provide construction company managers a valuable reference to benchmarking the CSR efficiency to help them find self-improvement ways to improve CSR efficiency performance.

## 1. Introduction

Over the past few decades, the construction industry has played a crucial role in the economic development of numerous countries [1]. However, it cannot be ignored that the economic boom driven by the construction industry has led to numerous environmental and social issues [2,3,4]. For example, as the second largest carbon emitter, the construction sector accounts for roughly 33% of global carbon emissions [5]. Furthermore, the construction sector, while employing only about 7% of the world’s workforce, it is responsible for 30–40% of fatal injuries worldwide [6]. The environmental and social pressures caused by the development of the construction industry are more prominent in China. For instance, though the construction industry has contributed to around 7% of the gross domestic product (GDP) and provided more than 30 million jobs in China [7], the construction industry energy consumption accounts for 20% of the total energy consumption of the country [8]. Given that those environmental and social issues pose a relatively massive threat to the sustainable development of the whole society [9,10,11,12], the government and company managers should pay increasing attention and take immediate measures to resolve this dilemma.

As a commitment to sustainable development, corporate social responsibility (CSR) has attracted attention from various business sectors since the mid-1900s [13], including the construction sector [14]. CSR is the responsibility of an organization for managing the impacts of its decisions and activities on the society and environment [15], which has been a widely used approach for responding to emerging social and environmental issues [16]. A large number of CSR principles (e.g., ISO26000, ISO14001, SA8000) have been developed by international organizations to encourage firm managers to formulate strategies to improve their social and environmental performance [17]. However, the CSR performance continues to be unsatisfactory in many industries, and the construction industry is no exception [18]. Transaction cost theory can explain this phenomenon very well, which proposed that efforts to improve CSR performance tend to serve to increase organizations’ financial burden [19,20]. For instance, construction companies are often involved in costly CSR activities related to the local community, such as providing free education or other philanthropic efforts, or assisting host countries with infrastructure construction such as roads, bridges or hospitals [21]. The financial burden caused by CSR will reduce the enthusiasm of construction companies to fulfill social responsibility, because neoclassical economics claims that if managers use company resources for any reason other than profit maximization, it will constitute a form of theft [22]. Given that CSR, as part of the company’s business operation [23], must receive resources (e.g., labor, capital) to fulfill, companies have begun to explore how to spend fewer resources to achieve higher CSR performance. As the degree to which resources are converted to CSR can be expressed in terms of efficiency [24], the challenge that companies face actually is how to achieve high CSR efficiency, which refers to the ratio between the low input of the organization’s resources and the high output of CSR performance.

There has been a considerable number of studies on efficiency. In these studies, the data envelopment analysis (DEA) model is a popular and practical method adopted to evaluate efficiency performance because it has advantages in terms of measuring efficiency performance based on multiple inputs and outputs without assigning prior weight to indicators [25,26]. Nowadays, the DEA model has been successfully applied in different fields, such as energy efficiency [27], education efficiency [28], policy efficiency [29], operational efficiency [30], investment efficiency [31] and urbanization efficiency [32]. Besides, after justifying DEA approach’s advantages in calculating CSR efficiency scores based on a comparison between DEA model and other approaches [33], a few CSR studies have attempted to adopt the DEA model to analyze CSR efficiency [34]. For example, Belu [24] analyzed CSR efficiency with the output-oriented DEA model in the industry sector, service sector, financial sector and technology sector. Furthermore, as benchmarking is considered to be an important way to improve efficiency performance [35], Yang [36] made an empirical case including 66 large firms in Taiwan with employing the two-objective DEA model and successfully identified benchmarking enterprises in terms of CSR efficiency.

However, one feature of these CSR efficiency studies is the neglect of environmental factors, which leads to not only the inaccuracy of the calculated efficiency score and thus the failure in identifying the benchmarking companies [37], but also the incapability in revealing the way that could improve CSR efficiency by controlling the environmental factors. To address the issue in regards to the inaccuracy of CSR performance caused by environmental variables, Fried and Lovell [38] proposed the three-stage DEA model which combines the DEA model proposed by Banker et al. [39] and the Stochastic Frontier Analysis (SFA) model proposed by Timmer [40]. The steps of three-stage DEA model includes: (1) utilization of BCC model to evaluate the efficiency performance and slacks of inputs or outputs; (2) use of SFA model to analyze the relation between environmental factors and efficiency performance to eliminate the impact of environmental factors; (3) re-estimation of the efficiency performance [41]. Nowadays, the three-stage DEA model has been widely employed in different industries, such as the transportation industry [42], the banking industry [43], the R&D industry [44], the manufacturing industry [45] and the construction industry [46].

To accurately evaluate CSR efficiency performance in the construction industry and thus identify ways to improve efficiency performance by setting benchmarking companies and controlling environmental factors, this study attempts to investigate the Chinese construction firms’ efficiency in fulfilling CSR by adopting the three-stage DEA to eliminate the effect of environmental factors. Specifically, objectives of this study are: (1) to use the DEA model to analyze the status of construction companies’ efficiency in fulfilling CSR; (2) to provide the government a scientific tool for targeted policy-making by adopting the SFA model to analyze the impact of environmental factors on CSR efficiency; (3) to provide self-improvement direction for construction companies to improve the management capacity of CSR efficiency by setting benchmarking companies.

The innovations and contributions of this article are to propose measures to reduce the cost of CSR in the construction industry from the perspective of efficiency, which is conducive to the construction enterprises to get rid of the financial burden and further improve the enthusiasm of fulfilling social responsibility. Specifically, results of this study present CSR efficiency performance of construction companies and reveal that the effect of marketization on CSR efficiency is complex and non-linear, which can be an empirical evidence for the government to understand the current state of CSR efficiency in the construction industry, as well as to formulate more targeted measures to promote construction companies’ efficient commitment of CSR. Furthermore, a valuable reference to benchmarking the “efficiency” of fulfilling CSR is provided to construction company managers, which can help them find self-improvement ways to improve their CSR efficiency. In addition, given that many construction companies from different countries are also facing the financial burden in fulfilling CSR, it is anticipated that the efficiency analysis of CSR in this study can be used by other governments and construction enterprises in different countries to understand the state quo of CSR efficiency in the local construction industry and provide local policy-makers and construction companies valuable references in policy-making and strategy-making to reduce the financial burden caused by CSR.

The remainder of the paper is organized as follows: following the Introduction, we briefly introduce in Section 2 the three-stage DEA model method. Section 3 describes the sample construction companies, variable and data used in the present study. Section 4 demonstrates the results and discussions from the empirical analysis. Section 5 sets forth conclusions and points out a number of potential shortcomings in the present study.

## 2. Methodology

The three-stage DEA model proposed by Fried and Lovell [38] is introduced to address the limitations of the DEA model which is widely used by existing CSR studies that cannot explain the impact of external institutional factors and internal management capabilities on efficiency, respectively. The steps of the three-stage DEA model are represented as follows:

### 2.1. Stage I: Output-Oriented BCC Model

Under the hypothesis that Variable Returns to Scale changes, Banker et al. [39] proposed a new DEA model to investigate efficiency, which is widely called the BCC model. The character of Variable Returns to Scale in the BCC model can eliminate the influences of scale factors and ensures that classifications of efficiency performance are invariant to the data transformation [47]. Furthermore, the BCC model can be divided into two types: input-oriented model and output-oriented model. To improve efficiency performance, the goal of the input-oriented DEA model is to decrease inputs when outputs are constant, and the goal of the output-oriented DEA model is to increase output when inputs are constant. As the purpose of CSR research is to maximize CSR performance, it is suitable to use the output-oriented BCC model for studying CSR efficiency [24]. The equation of the output-oriented BCC model is shown as follows:

(1) $$\begin{array}{c} {\mathrm{Max}}_{n}\;\theta \\ s.t.\overset{n}{\underset{i=1}{\sum }}\lambda_{i}X_{i}+s^{-}=X_{0}\\ \overset{n}{\underset{i=1}{\sum }}\lambda_{i}Y_{i}-s^{+}=\theta \;Y_{0}\\ \overset{n}{\underset{i=1}{\sum }}\lambda_{i}=1\\ \lambda_{i}\geq 0,\;i=1,\;2,\ldots ,\;n\\ s^{-},s^{+}\leq 0 \end{array}$$

where there are n Decision-Making Unit (DMU), and each DMU has m inputs and s outputs, Equation (1) can be used to calculate the efficiency of a particular DMU. $X_{i}={\left(x_{i1},\;x_{i2},\;\ldots ,\;x_{\mathrm{im}}\right)}^{T}$ refers to the input vector of DMU i; $Y_{i}={\left(y_{i2},\;y_{i2},\;\ldots ,\;y_{\mathrm{is}}\right)}^{T}$ refers to the output vector of DMU *j*; $\lambda_{i}$ represents the weight of DMU i; $s^{-}$ represents the slack variable of input; $s^{+}$ indicates slack variable of outputs; θ (0≤θ≤1) indicates the efficiency. In the practical operation, the efficiency is calculated by the DEA-SOLVER Pro 5.0 software. Before formal analysis with the output-oriented BCC model, Min-Max normalization should be performed on all data due to the fact that the DEA model is not compatible with non-positive data. And to avoid a value of 0, input and output data plus a positive value of 0.1 are used for data conversion. The feature of Variable Returns to Scale in the BCC model ensures that the classification of its efficiency and inefficiency is invariant to data conversion [47].

### 2.2. Stage II: SFA Model

The SFA model is adopted to analyze the effect of environmental variables on CSR efficiency. According to the existing studies [48,49], the SFA regression model can be expressed as:

(2) $$S_{\kappa i}=f^{\kappa }\left({Ζ}_{i}\;;\;\beta^{\kappa }\right)+\nu_{\kappa i}+\mu_{\kappa i};\;i\;=\;1,\;2,\;\ldots ,\;n;\;\kappa \;=\;1,\;2,\;\ldots ,\;m$$

in which $S_{\kappa i}$ refers to the kth output margin of DMU, $Z_{i}=\left(z_{1i},\;z_{2i},\;\ldots ,\;z_{\rho i}\right)$ is environment variables, where ρ is the number of environmental variables; $\beta^{\kappa }$ indicates the parameters to be estimated of the environment variables; $f^{\kappa }\left({Ζ}_{i};\;\beta^{\kappa }\right)$ is an indicator showing how the environment variable affects the output margin $S_{\kappa i}$; $\nu_{\kappa i}+\mu_{\kappa i}$ represents error the term. $\nu_{\kappa i}$ refers to random interference and is assumed to $\nu_{\kappa i}\;\sim \;Ν\left(0,\sigma_{\nu }^{2}\right)$; $\mu_{\kappa i}$ refers to the managerial inefficiencies, which is supposed to obey the truncated normal distribution, that is $\mu_{\kappa i}\;\sim \;{Ν}^{+}\left(\mu^{\kappa },\sigma_{\mu }^{2}\right)$.

According to Kumbhakar and Lovell [50], The estimator of $\mu_{\kappa i}$ can be obtained by using Equation (3):

(3) $$\hat{E}\left(\mu_{\kappa i}∕\nu_{\kappa i}+\mu_{\kappa i}\right){=\sigma }_{*}\left[\frac{\emptyset \left(\frac{{\lambda \varepsilon }_{\kappa }}{\sigma }\right)}{\Phi \left(\frac{{\lambda \varepsilon }_{\kappa }}{\sigma }\right)}+\frac{{\lambda \varepsilon }_{\kappa }}{\sigma }\right]$$

where $\sigma_{*}=\frac{\sigma_{\upsilon }\sigma_{\mu }}{\sigma }$, $\varepsilon_{i}=\nu_{\kappa i}+\mu_{\kappa i}$, $\sigma =\sqrt{\sigma_{\mu }^{2}+\sigma_{\nu }^{2}}$. ∅(·), Φ(·) are density function and distribution function of standard normal distribution, respectively. Therefore, the estimator of $\nu_{\kappa i}$ can be calculated by:

(4) $$E\left[\nu_{\kappa i}∕\nu_{\kappa i}+\mu_{\kappa i}\right]=S_{\kappa i}-f^{\kappa }\left({Ζ}_{i};\;\beta^{\kappa }\right)-\hat{E}\left(\mu_{\kappa i}∕\nu_{\kappa i}+\mu_{\kappa i}\right)$$

As such, the adjustment equation can be written as:

(5) $$x_{\kappa i}^{Α}=x_{\kappa i}+\left[\max\left(f^{\kappa }\left({Ζ}_{i};\;{\hat{\beta }}^{\kappa }\right)\right)\;-f^{\kappa }\left({Ζ}_{i};\;{\hat{\beta }}^{\kappa }\right)\right]+\left[\max\left({\hat{\nu }}_{\kappa i}\right)-\;{\hat{\nu }}_{\kappa i}\right];\;i=1,\;2,\;\ldots ,\;n;\;\kappa \;=\;1,\;2,\;\ldots ,\;m$$

where $x_{\kappa i}$ and $x_{\kappa i}^{Α}$ represent the output before and after the adjustment, respectively. $\mathrm{Max}\left(f^{\kappa }\left({Ζ}_{i};\;{\hat{\beta }}^{\kappa }\right)\right)-f^{\kappa }\left({Ζ}_{i};\;{\hat{\beta }}^{\kappa }\right)$ means adjusting external environmental factors and $\left[\max\left({\hat{\nu }}_{\kappa i}\right)-{\hat{\nu }}_{\kappa i}\right]$ represents the elimination of random errors. This study used the Frontier 4.1 software to run the SFA model.

### 2.3. Stage III: The Adjusted BCC Model

**Figure 1:**
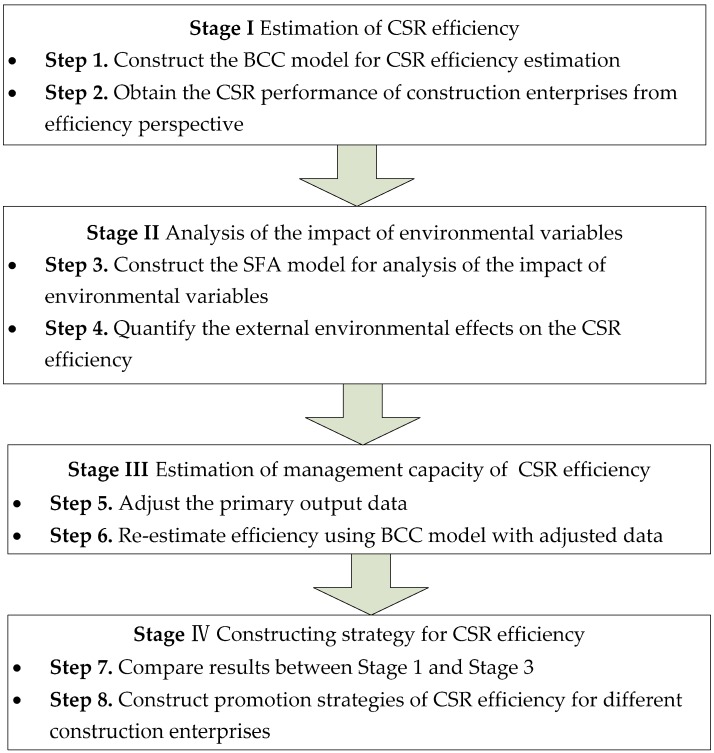
Flowchart of the proposed methodology.

In the third stage, the original input and adjusted output of each DMU are put into the BCC model to estimate the CSR efficiency management capacity. In combination with the above research steps, a methodological framework was systematically developed to illustrate the objectives and steps in each stage briefly. The methodology can be illustrated in the following stages: (1) to estimate the social performance of construction companies by using BCC model from efficiency perspective; (2) to analyze the impact of environmental variables on CSR efficiency by employing SFA model; (3) to estimate management capacity of CSR efficiency with the original input and adjusted output data by BCC model; and (4) to analyze and construct targeted strategies for promoting the CSR efficiency. The detailed diagrammatic description of the proposed methodological framework is shown in Figure 1.

## 3. Empirical Study

### 3.1. Sample Collection

Even though there are a large number of construction companies in China, this study selected those listed construction companies issued by China Securities Regulatory Commission [51] as samples, including 55 companies spanning the period of 2012–2016. Interpretively, from a financial perspective, the revenues of these 55 listed companies make up a significant proportion of the industry. For example, according to the report issued by Construction Times and Engineering News-Record [52], the sum of the revenues of the four listed construction companies (i.e., China State Construction Engineering, China Railway Construction, China Communications Construction and Shanghai Construction Group) contributed about half of total revenue of the top 80 Chinese contractors. From a regional perspective, these 55 listed companies come from different regions of China. Among the 55 listed construction companies, there are 43 companies from the eastern region, such as Zhejiang, Shanghai, Shandong, Jiangsu, Hainan, Guangdong and Beijing, with six from the western regions including Xinjiang, Xizang, Sichuan and Shanxi, and the other six are located in the central areas such as Hubei, Anhui and Heilongjiang. Additionally, the disclosed information (e.g., financial data) of listed companies is more reliable [53]. These enable these 55 enterprises to truly reflect the status quo of the construction industry in China. What’s more, although Shanghai Stock Exchange promulgated regulations on strengthening the social responsibility of the listed companies in 2008, the Ministry of Commerce did not issue the CSR standards of contractors until 2012 [54], which is the reason why the sample collection in this study begins in 2012. The list of the studied companies is shown in Appendix A.

### 3.2. Input Variable and Output Variable

CSR is widely considered to encompass four types of responsibility (i.e., economic responsibility, legal responsibility, ethical responsibility, and philanthropic responsibility), where economic responsibility is the most basic one and account for a large proportion of CSR [55,56]. As an important way to achieve economic responsibility, business activities are regarded as the most significant approach for enterprises to undertake their social responsibility. That is to say, CSR is a by-product of business operations [57,58,59]. Therefore, resources invested by enterprises in their business activities can be regarded as resources invested in CSR. In addition, compared with business activities, few resources are invested in other activities (e.g., ethical and charitable activities). For instance, charitable donations from Chinese top 500 charity enterprises account for only 0.0348% of their total revenue [60]. Thus, this study selected the resources which are put into business activities as inputs for fulfilling CSR.

The business operation of any company requires an investment of labor, capital and equipment, and construction firms are no exception [46,61]. Table 1 shows the measurement methods for the variables of labor, capital and equipment in the construction industry. Given that the current ratio indicator reflects the companies’ short-term financial condition, and the indicators of debt-to-equity ratios and growth in the cost of goods sold indicate the long-term financial status, these three indicators were adopted to represent the company’s capital resource. In addition, because salaries expense can be regarded as the cost of the labor force, this paper used salaries expense to represent labor resource. Similarly, the value of machinery and equipment can also be deemed as the expense of the company’s equipment [62], supporting that the value of machinery and equipment per revenue can represent the equipment resource. The data for input indicators were acquired from the company’s annual report.

According to previous studies [73,74,75], the output indicators chosen for evaluating the CSR performance always involve multiple stakeholders including shareholders, employees, suppliers, clients, customers, environment, and community. To measure the CSR performance of construction companies, this study adopted a listed company database developed by Hexun. As a former subsidiary organization of the China Securities Market Research and Design Center, Hexun has good cooperation with Thomson Reuters and the Shanghai Stock Exchange and provides services for more than 60 million users monthly on average and more than 100 million annually [76]. Unlike other agency-based databases including Kinder, Lydenberg & Domini (KLD), Fortune, the Dow Jones Sustainability Index (DJSI) and Canadian Social Investment, CSR database developed by Hexun focuses on Chinese listed companies. The original data of Hexun are derived from the company’s annual report or CSR report. Nowadays, CSR database developed by Hexun has been proven to be reliable in many industries [74,77,78]. Therefore, Hexun’s CSR database was adopted in this study. Table 2 lists the descriptive statistics for the selected input and output indicators.

### 3.3. External Institutional Environmental Factors

In general, institutional environmental variables are factors that influence the CSR efficiency but are out of control of the construction companies. Existing studies have already demonstrated the relevance between governmental behavior and corporate social responsibility. For instance, Matten and Moon [79] proposed that the government’s economic intervention largely determines the types of CSR issues to which corporations respond. Wu [80] also indicated that the government intervention is one of the dominant factors influencing CSR behavior. Given that marketization could reflect the government behavior in the market [81] and has a profound effect on other institutional factors (e.g., economy, society, law, and political system) [82], this study focused on the institutional variables of the marketization.

Marketization is characterized by five indicators including (1) the relationship of intervention between the government and market; (2) the development of the non-state economy; (3) the development of the product market; (4) the development of the factor market; and (5) the development of the legal-system environment [81]. The data of indicators were obtained from the database developed by the National Economic Research Institute, which is widely applied for measuring the maturity of marketization in Chinese provinces [83]. However, the latest full version of this database only contains data from 2008–2014 [81], which does not match the period of 2012–2016 in this research. Therefore, it is necessary to estimate the index of 2015 and 2016 based on the 7-year data from 2008–2014. In recent years, China’s marketization has developed steadily. A strong evidence is that China has consistently maintained a steady GDP growth rate, which is mainly driven by marketization [84]. Therefore, China’s marketization index has stability in time series, which can ensure the valid prediction of using 7-year data. In view of the existing research indicated that the exponential smoothing method is an effective method to predict data index of marketization [85,86], this study adopted the exponential smoothing method to measure the index of 2015 and 2016 based on the 7-year data from 2008–2014. Table 3 shows the descriptive statistics of external environmental factors.

## 4. Results and Discussion

### 4.1. Stage I: Efficiency Based on BCC Model

**Figure 2:**
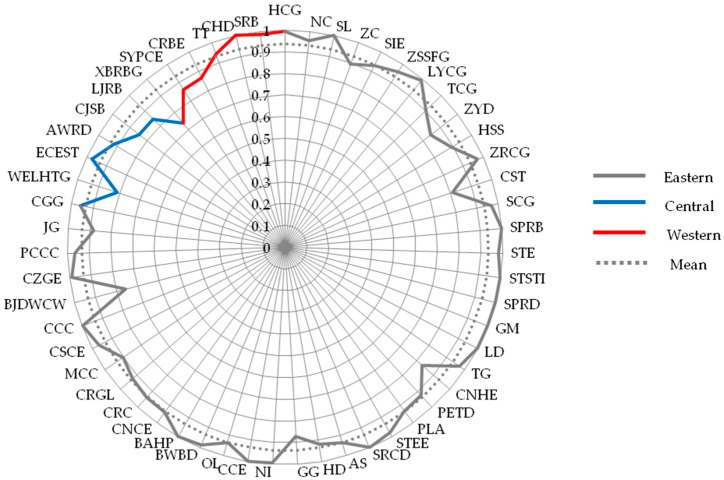
Average efficiency score of each firm in stage I.

In stage I, the BCC model based on Equation (1) was used to calculate the annual efficiency scores of samples during 2012–2016, which is shown in Appendix B. However, in view of the contingency of cross-sectional data, the average scores of these construction companies during the 2012–2016 period were used to investigate the overall CSR efficiency performance, which reflects the robustness of construction companies to efficiently undertake their social responsibilities. These results are shown in Figure 2.

In DEA model, firms located on the efficient frontier are considered to be the most efficient company in the group, whose efficiency scores are 1, and the efficiency score represents the ratio of the company’s efficiency performance to the best efficiency performance within the group [36,39]. Specifically, with respect to our research, those construction companies with the score of 1 have achieved the best performance in CSR efficiency compared with other samples. This means that these firms can undertake the highest level of CSR when consuming the same resources relative to other firms. Firms with the CSR efficiency value of 0.935 imply their performance is 0.935 times that of companies with a score of 1. Overall, Figure 2 demonstrates that the average CSR efficiency score was 0.935 among the 55 listed construction companies and only ten companies achieved the optimal level of CSR efficiency during 2012–2016, such as China Camc Engineering Company and Shanghai Pudong Road and Bridge Company. This shows that most construction companies’ CSR efficiency can be improved, implying that there is still a significant gap in the listed construction companies’ efficiency of fulfilling social responsibilities.

**Figure 3:**
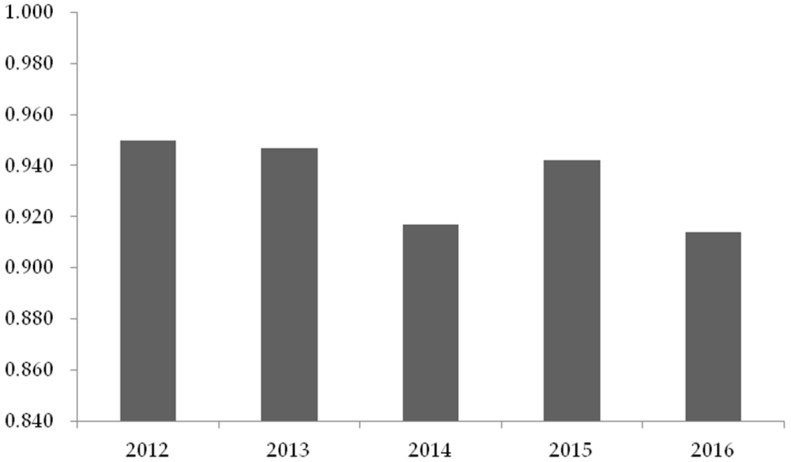
Average efficiency score of each year in stage I.

In addition, Figure 3 displays the average efficiency score of each year in the stage I. It shows that the average annual efficiency score has dropped from 0.950 in 2012 to 0.914 in 2016, with an overall downward trend. One important reason explaining this downward trend could be the continuous development of the legal-system environment. For instance, the CSR standards for contractors were promulgated in 2012 [54]. Therefore, it is necessary to explore the impact of the development of the legal-system environment on the CSR efficiency in stage II, to analyze the reasons for the change of CSR efficiency during 2012–2016.

From the regional perspective, it was found that the overall efficiency of the listed construction companies in different regions (i.e., the eastern region, the central region, and the western region) has not reached an optimal level. 

**Figure 4:**
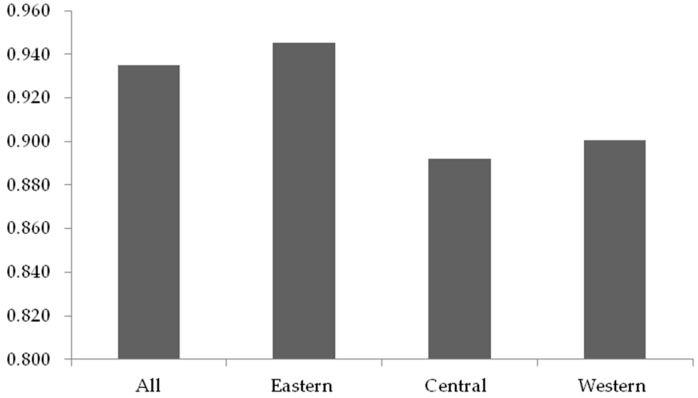
Mean efficiency for each region in stage I.

As shown in Figure 4, the average score of the listed construction companies in the eastern region is the highest in all regions. The efficiency scores of the construction companies in the central and western regions are close to each other. Nevertheless, they are both lower than the average score. The differences among areas are likely to be attributed to the external circumstances they faced. As proposed by Liu et al. [87], the proportion of non-state-owned construction companies in the eastern region is higher than companies in the central or the western region. Therefore, it is also essential to explore the impact of the development of the non-state economy on the CSR efficiency in stage II, so as to analyze the reasons for the difference of CSR efficiency in regions.

In addition, setting optimal companies as benchmark companies is one of the practical ways to improve a company’s CSR efficiency. Low-efficiency companies can learn CSR management methods from high-efficiency companies to strengthen their management capacity of CSR efficiency. However, it is worth noting that the efficiency of companies will be overestimated in the favorable circumstance [37]. Therefore, it is also necessary to eliminate the influence of the external institutional environment on CSR efficiency and re-evaluate the efficiency of construction companies.

### 4.2. Stage II: Using SFA to Quantify Environmental Effects

Based on the SFA model, the impact of environment on CSR is quantified. However, the relation between the development of the factor market and CSR efficiency is not significant because its t-values for each variable were −0.114, −0.670, 0.450, −0.334, and 0.383 respectively. Therefore, the development of the factor market was removed and the remaining variables of marketization were again subject to SFA analysis. Table 4 shows the regression results.

The results in Table 4 indicate that the relationship of intervention between the government and market is negatively related to the SESR, SSCCR, SER and SCR. This means that the decrease of government intervention in the market contributes to the decrease of SESR, SSCCR, SER and SCR, which could improve the CSR efficiency. Corruption is more likely to occur in the construction industry in the context of high government intervention [88], and firms are likely to adopt the rent-seeking behavior rather than the corporate environmental and charitable behavior to obtain more benefits in corrupt context [89], leading to the company’s lack of incentive to improve the CSR efficiency. Therefore, to improve CSR efficiency of construction companies, the government should weaken its intervention in the market.

Besides, the development of the non-state economy is also negatively related to SSR, SESR, SSCCR, SER and SCR, indicating that the growth of the non-state economy is conducive to improving the efficiency of construction companies in fulfilling CSR. In China, state-owned companies not only occupy a dominant position among listed companies [90], but also hold a significant share in the construction market [87], which implies that non-state-owned companies have less resource to peruse CSR compared with state-owned companies. In order to undertake more CSR than state-owned companies to enhance the competitiveness, non-state-owned companies have the higher motivation to improve CSR efficiency, which in turn gives state-owned companies the incentive to enhance their CSR efficiency because they need to maintain a competitive advantage in the industry. In addition, since the development of the non-state economy has a positive effect on CSR efficiency, it is deemed that the growth of the non-state economy is an essential factor for the difference of CSR efficiency among regions in Stage I.

Moreover, the development of the product market shows a positive relationship with SSR, SESR, SSCCR, SER and SCR, which implies that the growth of product market reduces the CSR efficiency of construction companies. Currently, most consumers are unwilling to pay more to buy products from more socially responsible companies in China [91], indicating that it is hard for companies to gain benefit from socially responsible activities. With the development of the product market, the price of products tends to be determined by the market due to the high level of market competition [81], and therefore it becomes increasingly difficult for construction companies to improve their financial performance by undertaking CSR. As firms refuse to engage in socially responsible practices under the condition where profit is not assured [92], construction companies are likely to give up improving their CSR performance and CSR efficiency with the development of the product market owing to low profit in CSR.

Furthermore, the development of the legal-system environment also presents a positive relationship with all slacks of input, which implies that the development of the legal-system climate is not conducive to improving the construction companies’ CSR efficiency. This is mainly due to the fact that CSR efficiency depends on both the input of resources and output of CSR performance. With the development of the legal-system environment, the output of CSR performance will improve. However, it cannot be ignored that the CSR standard will also develop along with the development of the legal-system environment, which makes companies need to spend more resources to fulfil than before. For example, initial learning costs are unavoidable for companies facing a new standard of social responsibilities [93], which relies on the huge investment of resource. Therefore, under the context of large resource invested in satisfying new CSR standard, CSR efficiency performance is likely to go down though the output of CSR performance increases with the development of the legal-system environment. Meanwhile, as the development of the legal-system affects CSR efficiency negatively, the development of the legal-system environment is an essential factor contributing to the downward trend of CSR efficiency during 2012–2016 in Stage I.

Through the above analysis, the results show that the effect of marketization on CSR efficiency is complex and non-linear. In the process of promoting marketization, the Chinese government can improve the CSR efficiency of construction companies by reducing the government intervention in the market and supporting the development of the non-state economy. However, due to the adverse effect of the development of the product market and the legal-system environment on the CSR efficiency, it is relatively imperative for the government to adopt more targeted measures to alleviate this dilemma. For instance, it is encouraged that CSR can be chosen by the construction companies as a selling point to improve the benefits associated with social responsibility. In addition, with the development of relevant legal system, the government should enhance the CSR experience of construction companies by providing CSR training, etc., thereby improving the CSR efficiency of construction companies.

In addition, these results and methodologies can also be used as a reference for other developing countries. For example, as the emerging powers in the world, China and South Africa are unsustainable in the urbanization processes [94], which urgently need construction companies to fulfill CSR to address social and environmental issues. Therefore, the government in South Africa also can improve CSR efficiency of construction companies by regulating marketization based on the analysis of the relation between marketization and CSR efficiency with the three-stage DEA model, and thus increase sustainability performance in the urbanization processes.

### 4.3. Stage III: Re-Estimate Efficiency Using Adjusted Data

**Figure 5:**
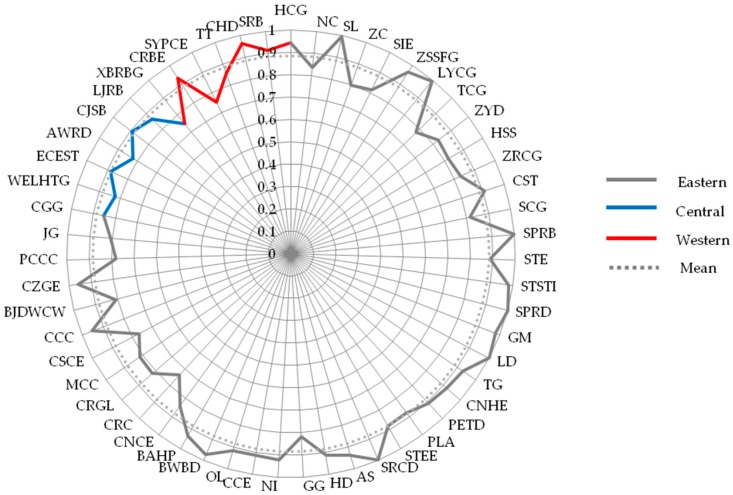
Average efficiency score after eliminating the institutional variable and random factor.

In Stage III, the BCC model based on original inputs and adjusted outputs was employed to recalculate and interpret the CSR efficiency score. The efficiency score of samples in each year are presented in Appendix B. Furthermore, to overcome the shortcoming of the contingency of cross-sectional data, the average scores of these construction companies during the 2012–2016 period are evaluated, which are shown in Figure 5.

As shown in Figure 5, the average efficiency of construction companies has not reached the optimum as the corresponding score is 0.884, and only a few construction companies have achieved the best efficiency. As suggested by Liu and Liu [37], efficiency values will veritably reflect the company’s management capacity without environmental disturbances, which implies that the efficiency value in stage III represents management capacity of CSR efficiency in the construction industry. Figure 5 shows that there is a gap in the management capacity of CSR efficiency of construction companies, indicating that setting up benchmarking companies is a practical way to improve the management capacity of CSR efficiency in the Chinese construction industry.

**Figure 6:**
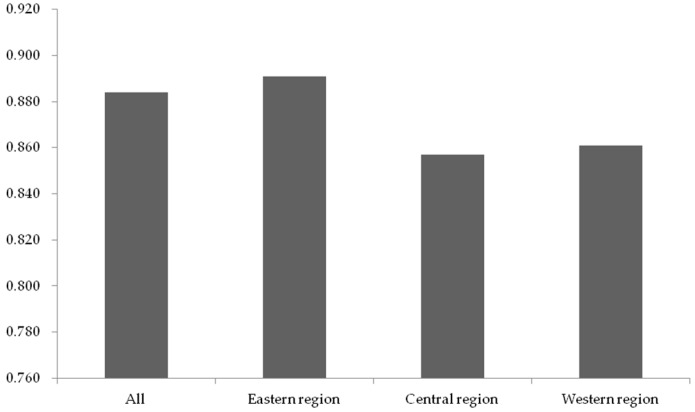
Mean efficiency for each region in stage III.

In addition, as shown in Figure 6, the construction company’s management capacity of CSR efficiency in each region has not reached the optimal level and gaps of management capacity of CSR efficiency exist among different regions, where efficiency score in the eastern region is the highest. Therefore, the government should strengthen cooperation among construction companies from the different areas, which is beneficial to the transmission of knowledge of CSR management among companies.

### 4.4. Comparison of Results between Stage I and Stage III

In order to identify the problems of CSR efficiency encountered by the Chinese construction companies and construct a comprehensive strategy for improving the CSR efficiency, it is necessary to investigate the different performance levels before and after eliminating the impact of external factors. Table 5 shows the descriptive statistics of CSR efficiency obtained in Stage I and the efficiency scores analyzed from Stage III which removes the institutional influences. From the time level, it can be found that the efficiency score in Stage I shows a downward trend during 2012–2016 while the efficiency score in Stage III that represents the CSR management capacity maintains a stable trend. This shows that the deterioration of the external institutional environment is an essential reason for the decline in the efficiency score of construction companies during this period. To eliminate the deterioration of the external institutional environment, government intervention in the market should be reduced gradually and the development of the non-state economy can be supported to further improve the CSR efficiency. Moreover, the government should take measures such as encouraging the market to adopt CSR as a selling point and providing CSR training to control the adverse effects of the development of the product market and legal system environment. Besides, Table 5 shows that after eliminating the impact of the external environment, the efficiency of listed construction companies in the different regions has declined. This shows that the institutional environment in different regions effectively affects the efficiency of construction companies in fulfilling CSR.

Also, from the corporate level, Figure 2 and Figure 5 show that the average efficiency scores have declined after controlling for the impact of the external environment. The drop in efficiency score confirms that construction companies are more likely to achieve optimal CSR efficiency in a favorable external institutional environment. Meanwhile, it is worth noting that gaps may exist in some companies’ efficiency scores between Stage I and Stage III. For example, some companies with high efficiency scores in the Stage I have not achieved high scores in Stage III, such as Shanghai Construction Group and China Gezhouba Group. Therefore, it is necessary to analyze the gap of the efficiency score of each company and explore an accurate way to improve their CSR efficiency.

As average CSR efficiency score reflects the average level of CSR efficiency of samples, firms with above-average scores can be considered to undertake CSR efficiently. Therefore, according to the scores of construction companies in Stage I and Stage III, the listed construction companies are divided into four types based on the critical point of the company’s overall average value. The first type is double-high type of companies whose efficiency scores are high in both Stage I and Stage III, including Shanghai Pudong Road Company and Bridge and China Communications Construction Company. Because these companies can maintain high efficiency scores before and after eliminating the environmental impacts, they are qualified to act as benchmarks for other construction companies. The second type is high-low companies which have a high score in Stage I and low score in Stage III, such as Shanghai Construction Group and China Gezhouba Group. After eliminating the impact of the institutional environment, the efficiency score of the second type of companies has dropped significantly, which implies that the high efficiency of such companies depends on a favorable external institutional environment. The measure to increase the efficiency of the second type of companies is to improve their management capacity of CSR efficiency. The third type is double-low type of companies whose efficiency scores are low in Stage I and Stage III, such as Anhui Water Resources Development Company and Xinjiang Beixin Road and Bridge Group. The increase of the efficiency for the third type of companies depends on not only the improvement of the institutional environment, but also the enhancement of management capacity for CSR efficiency. Contrary to the second type of company, the fourth type is low-high companies which have a low score in Stage I and a high score in Stage III, such as Guangdong No. 2 Hydropower Engineering Company and Chengdu Road and Bridge Engineering Company. The CSR efficiency of construction companies can be improved after controlling the impact of the institutional environment, which indicates that improving the institutional environment is the best way for the third type of companies to increase the CSR efficiency.

**Figure 7:**
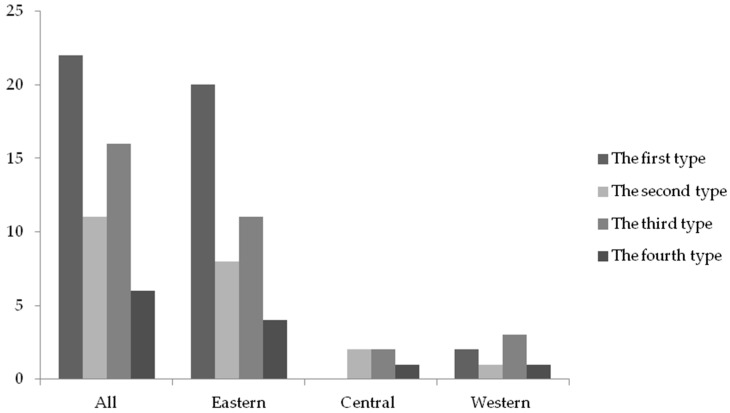
Distribution of company types by region.

Furthermore, the distribution of these four types of companies by region is shown in Figure 7. It is evident that the proportion of the first type of companies is less than half and the proportion of the second types of companies is only lower than that of the first type, which implies that, currently, the improvement of the CSR efficiency in the Chinese construction industry relies not only on optimizing the institutional environment, but also on enhancing management capacity of CSR efficiency. In addition, because most of the first type of companies locate in the eastern region, the government should strengthen the cooperation between the first type of companies and construction companies from different regions, which can promote the diffusion of CSR practice in the industry and enable the first type of firms to fully play the role of the benchmarking for other companies.

## 5. Conclusions

The more efficient in fulfilling CSR, the fewer resources companies need to invest. This allows companies to release more resources to focus on their main business, and therefore the issue of CSR efficiency has become increasingly important. In this study, 55 listed construction companies issued by the China Securities Regulatory Commission were selected as samples, and the three-stage DEA model was adopted to analyze the CSR efficiency with a focus on the Chinese construction industry. The results show that the average CSR efficiency score is 0.935 among the 55 listed construction companies during the 2012–2016 period while that drops to 0.884 after eliminating the effects of environmental factors, where more than half of the companies have performance below the average. From the regional perspective, there are differences among areas regarding average CSR efficiency performance of construction companies, and the majority of the companies with high CSR efficiency performance locate in the eastern region. These findings can serve as a basis for stakeholders to understand the status quo of CSR efficiency in the construction industry, and help them identify benchmarking firms, so as to help construction companies with inefficient CSR performance find self-improvement ways to enhance the CSR management capacity. In addition, the empirical results also show that both the decrease of government intervention in the market and the growth of the non-state economy contribute to the improvement of CSR efficiency. On the contrary, the development of the product market and the legal system have a negative effect on the CSR efficiency. This discovery enables the government to better understand the relation between marketization and CSR efficiency in the construction industry, and therefore provides a useful reference for the government to take measures in controlling the adverse effects of marketization.

However, there are still some limitations in this study which need to be further studied and responded to in the future. First, this paper used the CSR database developed by Hexun. However, the evaluation and measurement of CSR are still controversial, and the results may be different if another database is used. Therefore, a more reliable and authoritative indicator system for measuring CSR should be developed in the future. Second, the data for this empirical study are mainly collected from the year of 2012 to 2016 for those listed construction companies, which is primarily limited by the data availability. Future studies could continue to extend to more construction companies for a longer period of time.

## Figures and Tables

**Table 1:** Summary of Variables Selection.

Proxies	Variables Selection	Literature
Capital resources	Current ratio	[63,64,65]
	Debt to equity ratio	[64,66,67]
	Growth ratio in the cost of goods sold	[65,68]
Labor resources	Growth ratio of salaries expense	[69,70,71]
Equipment resource	Value of machinery and equipment/revenue	[62,72]

**Table 2:** Means (and standard deviations) of input and output factors.

Variables	2012	2013	2014	2015	2016
Current ratio	1.518	1.460	1.341	1.438	1.427
	(1.011)	(0.848)	(0.496)	(0.544)	(0.422)
Debt to equity ratio	2.867	3.082	3.261	2.720	2.678
	(2.203)	(2.512)	(3.319)	(1.841)	(1.973)
Growth ratio in the cost of goods sold	0.150	0.240	0.116	0.051	0.117
	(0.240)	(0.476)	(0.260)	(0.279)	(0.336)
Growth ratio of salaries expense	0.521	0.585	0.179	0.138	0.009
	(1.788)	(1.725)	(0.309)	(0.422)	(1.197)
Value of machinery and equipment/revenue	0.064	0.061	0.640	0.046	0.040
	(0.104)	(0.115)	(0.120)	(0.072)	(0.067)
shareholders	15.082	15.158	14.119	14.040	13.029
	(5.751)	(6.288)	(4.514)	(4.609)	(5.250)
Employees	5.755	5.507	2.902	4.060	4.556
	(5.188)	(5.012)	(2.797)	(4.605)	(4.862)
Suppliers, clients and consumers	3.636	4.655	0.691	2.309	3.018
	(5.107)	(6.263)	(2.538)	(5.059)	(5.579)
Environment	6.073	6.306	0.818	3.427	3.864
	(8.529)	(8.570)	(3.339)	(7.481)	(7.378)
Community	4.556	4.406	3.986	3.866	4.281
	(3.717)	(3.942)	(4.974)	(3.496)	(4.474)

**Table 3:** Means (and standard deviations) of external environmental factors.

Variables	2012	2013	2014	2015	2016
Relationship of intervention between government and market	6.675	6.629	6.949	6.443	6.233
	(2.032)	(2.171)	(2.20)	(2.477)	(2.661)
Development of the non-state economy	7.766	8.097	8.384	8.530	8.751
	(1.717)	(1.773)	(1.643)	(1.676)	(1.689)
Development of the product market	7.661	7.661	7.462	7.539	7.519
	(1.366)	(1.366)	(1.807)	(4.605)	(1.658)
Development of the factor market	7.092	7.578	8.388	8.714	9.224
	(2.807)	(2.966)	(3.032)	(3.384)	(3.651)
Development of the legal-system environment	10.198	10.663	11.872	12.637	13.531
	(4.028)	(4.105)	(4.121)	(4.774)	(5.289)

**Table 4:** Means (and standard deviations) of external environmental factors.

Variables	SSR	SESR	SSCCR	SER	SCR
Constant term	−0.029	0.118	0.277 **	0.159 *	−0.016
Relationship of intervention between government and market	0.014	−0.311 **	−0.508 ***	−0.328 **	−0.012
Development of the non-state economy	−0.105 *	−0.126	−0.156	−0.046	−0.076
Development of the product market	0.088 **	0.230 ***	0.318 ***	0.165 **	0.072 **
Development of the legal-system environment	0.072 *	0.227 ***	0.250 ***	0.158 **	0.059 **
σ^2^	0.012	0.065	0.101	0.035	0.010
γ	0.354	0.536	0.511	0.327	0.5433
LR test of the one-sided error	26.720	22.946	16.694	8.969	28.097

SSR: Slacks of shareholders responsibility; SER: Slacks of employees responsibility; SSCCR: Slacks of suppliers, clients and consumers responsibility; SER: Slacks of environment responsibility; SCR: Slacks of community responsibility; *** indicates significant at 1% level; ** indicates significant at 5% level; * indicates significant at 10% level.

**Table 5:** The results of Stage I and Stage III.

Factor	Time Level					Region Level		
	2012	2013	2014	2015	2016	Eastern	Central	Western
Stage I	0.950	0.947	0.917	0.945	0.914	0.945	0.902	0.900
Stage III	0.875	0.877	0.892	0.903	0.873	0.891	0.856	0.861

## Appendix A

**Table A1:** Summary of the sample.

No.	Name	No.	Name
1	Tagen Group (TG)	29	Long Jian Road and Bridge (LJRB)
2	ChengDu Hi-Tech Development (CHD)	30	Sinoma International Engineering (SIE)
3	Zhongnan Construction (ZC)	31	China National Chemical Engineering (CNCE)
4	China Camc Engineering (CCE)	32	China Railway Construction (CRC)
5	Norinco International (NI)	33	China Railway Group Limited (CRGL)
6	Guangdong No.2 Hydropower Engineering (CNHE)	34	Metallurgical Corporation of China (MCC)
7	Hongrun Construction Group (HCG)	35	China State Construction Engineering (CSCE)
8	Zhejiang Southeast Space Frame Group (ZSSFG)	36	China Communications Construction (CCC)
9	East China Engineering Science and Technology (ECEST)	37	Gold Mantis(GM)
10	Xinjiang Beixin Road and Bridge Group(XBRBG)	38	Avic Sanxin (AS)
11	Orient Landscape (OL)	39	Hongtao Decoration (HD)
12	Shandong Lipeng (SL)	40	Zhejiang Yasha Decoration (ZYD)
13	Palm Eco-Town Development (PETD)	41	Grandland Group (GG)
14	Beijing Water Business Doctor (BWBD)	42	Beijing Jiayu Door Window and Curtain Wall (BJDWCW)
15	Sichuan Road and Bridge(SRB)	43	Shanghai Prosolar Resources Development (SPRD)
16	China Gezhouba Group (CGG)	44	Lowton Development (LD)
17	CSSC Science and Technology (CST)	45	Hanggang Steel Structure (HSS)
18	Wuhan East Lake High Technology Group(WELHTG)	46	Changjiang and Jinggong Steel Building (CJSB)
19	Shanghai Construction Group (SCG)	47	China Zhonghua Geotechnical Engineering (CZGE)
20	Shaanxi Yanchang Petroleum Chemical Engineering (SYPCE)	48	Zhejiang Reclaim Construction Group (ZRCG)
21	Shanghai Pudong Road and Bridge(SPRB)	49	Chengdu Road and Bridge Engineering (CRBE)
22	Tibet Tianlu (TT)	50	Pubang Landscape Architecture (PLA)
23	Beijing Airport High-tech Park (BAHP)	51	Shenzhen Techand Ecology and Environment (STEE)
24	Long Yuan Construction Group (LYCG)	52	Power Construction Corporation of China (PCCC)
25	Anhui Water Resources Development (AWRD)	53	Ningbo Construction (NC)
26	Tengda Construction Group (TCG)	54	Shenzhen Ruihe Construction Decoration (SRCD)
27	Shanghai Tunnel Engineering (STE)	55	Jangho Group (JG)
28	Shanghai Tongji Science and Technology Industrial (STSTI)		

## Appendix B

**Table A2:** The scores of CSR efficiency of the Stage I and Stage III in years 2012–2016.

Name	Stage I					Stage III				
	2012	2013	2014	2015	2016	2012	2013	2014	2015	2016
TG	0.851	1.000	1.000	1.000	1.000	0.899	0.952	1.000	1.000	0.791
CHD	1.000	1.000	1.000	1.000	1.000	1.000	0.819	1.000	1.000	1.000
ZC	0.883	1.000	0.888	1.000	0.700	0.810	0.829	0.732	0.945	0.681
CCE	1.000	1.000	1.000	1.000	1.000	0.884	0.896	0.819	0.998	0.964
NI	1.000	1.000	1.000	1.000	0.965	1.000	1.000	1.000	1.000	0.606
CNHE	0.721	0.866	0.705	0.862	1.000	0.795	0.982	0.874	0.997	0.941
HCG	1.000	0.958	1.000	1.000	1.000	0.942	0.980	0.881	0.995	0.913
ZSSFG	1.000	0.964	1.000	1.000	0.837	1.000	0.962	1.000	1.000	0.863
ECEST	0.963	1.000	0.902	1.000	1.000	0.965	0.732	0.720	1.000	1.000
XBRBG	0.746	0.750	0.618	0.769	0.810	0.831	0.610	0.699	0.909	0.698
OL	1.000	1.000	1.000	0.832	0.860	0.764	1.000	1.000	1.000	0.826
SL	1.000	1.000	1.000	1.000	1.000	1.000	0.978	1.000	1.000	1.000
PETD	1.000	1.000	0.923	0.703	1.000	0.709	1.000	0.826	1.000	1.000
BWBD	1.000	1.000	1.000	0.931	1.000	0.900	1.000	0.970	1.000	1.000
SRB	1.000	0.936	1.000	1.000	1.000	0.801	0.831	1.000	1.000	0.931
CGG	0.968	0.974	0.964	0.950	0.935	0.811	0.933	0.763	0.982	0.774
CST	1.000	0.843	0.830	0.883	0.494	1.000	0.867	0.942	1.000	0.738
WELHTG	1.000	0.305	0.755	1.000	1.000	0.878	0.710	0.669	1.000	0.873
SCG	1.000	1.000	0.931	1.000	0.905	0.812	0.914	0.609	1.000	0.740
SYPCE	0.768	0.781	0.964	1.000	0.800	0.652	0.749	0.655	1.000	0.713
SPRB	1.000	1.000	1.000	1.000	1.000	1.000	1.000	1.000	1.000	1.000
TT	0.911	1.000	1.000	0.903	0.912	1.000	1.000	1.000	0.377	0.901
BAHP	1.000	1.000	1.000	1.000	1.000	1.000	1.000	1.000	0.777	0.891
LYCG	1.000	1.000	0.954	1.000	1.000	1.000	1.000	0.967	1.000	1.000
AWRD	1.000	1.000	0.803	1.000	0.775	1.000	1.000	0.925	0.709	0.486
TCG	0.793	1.000	0.760	0.958	1.000	0.504	0.675	0.708	1.000	1.000
STE	1.000	1.000	0.928	0.982	1.000	1.000	0.603	0.840	1.000	1.000
STSTI	1.000	1.000	1.000	1.000	1.000	0.999	0.998	0.968	1.000	0.934
LJRB	0.836	1.000	0.656	0.950	0.789	0.731	1.000	0.873	0.963	0.746
SIE	1.000	1.000	0.661	1.000	1.000	0.811	0.744	0.868	0.836	0.812
CNCE	0.862	0.998	0.913	0.925	1.000	0.860	0.699	0.753	0.921	0.988
CRC	1.000	1.000	0.889	0.897	0.914	0.867	0.610	0.645	0.642	0.909
CRGL	0.998	0.982	0.826	0.894	0.922	0.916	0.794	0.700	0.860	0.812
MCC	1.000	1.000	0.857	0.907	0.726	0.891	0.820	0.705	0.819	0.856
CSCE	1.000	0.942	0.999	0.946	0.933	0.849	0.660	0.743	0.753	0.818
CCC	1.000	1.000	0.982	1.000	1.000	0.758	1.000	1.000	1.000	1.000
GM	1.000	1.000	1.000	1.000	1.000	1.000	1.000	0.905	1.000	1.000
AS	0.730	0.955	1.000	1.000	1.000	0.690	1.000	1.000	1.000	1.000
HD	1.000	1.000	1.000	0.881	0.718	0.800	1.000	1.000	1.000	0.768
ZYD	0.860	0.654	0.975	1.000	0.741	0.590	0.841	0.924	1.000	0.791
GG	0.910	0.891	0.950	0.806	0.798	0.912	0.706	0.951	0.791	0.741
BJDWCW	0.872	0.771	0.667	0.748	0.726	0.876	0.716	0.921	0.706	0.822
SPRD	1.000	1.000	1.000	1.000	1.000	1.000	1.000	1.000	1.000	1.000
LD	1.000	1.000	1.000	1.000	1.000	1.000	1.000	1.000	1.000	1.000
HSS	0.834	0.881	0.799	0.999	0.949	0.681	0.733	0.938	0.999	0.745
CJSB	0.927	0.947	0.767	0.720	0.886	0.687	0.969	0.982	0.828	1.000
CZGE	1.000	1.000	1.000	0.990	0.955	1.000	1.000	1.000	0.897	0.895
ZRCG	0.984	0.935	0.946	1.000	1.000	0.665	0.876	0.945	0.678	1.000
CRBE	1.000	1.000	1.000	0.855	0.484	1.000	1.000	1.000	0.935	0.723
PLA	1.000	1.000	1.000	0.865	0.807	1.000	1.000	1.000	0.634	0.757
STEE	1.000	1.000	0.999	0.895	1.000	1.000	0.734	1.000	0.739	0.943
PCCC	1.000	1.000	0.928	0.908	1.000	0.914	0.710	0.937	0.489	0.861
NC	0.957	0.989	0.926	1.000	0.917	0.859	0.795	0.857	0.837	0.840
SRCD	1.000	1.000	1.000	1.000	1.000	1.000	1.000	1.000	1.000	1.000
JG	0.882	0.776	0.761	1.000	1.000	0.837	0.788	0.824	0.653	0.931

## References

[1] Xue H., Zhang S.J. (2017). Relationships between engineering construction standards and economic growth in the construction industry: The case of China’s construction industry. KSCE J. Civ. Eng..

[2] Chang R.D., Zuo J., Soebarto V., Zhao Z.Y., Zillante G., Gan X.L. (2017). Discovering the Transition Pathways toward Sustainability for Construction Enterprises: Importance-Performance Analysis. J. Constr. Eng. Manag..

[3] Lu W., Ye M., Flanagan R., Ye K. (2016). Corporate Social Responsibility Disclosures in International Construction Business: Trends and Prospects. J. Constr. Eng. Manag..

[4] Shen L.Y., Tam V.W.Y., Tam L., Ji Y.B. (2010). Project feasibility study: The key to successful implementation of sustainable and socially responsible construction management practice. J. Clean. Prod..

[5] Wu Y., Chau K.W., Lu W., Shen L., Shuai C., Chen J. (2018). Decoupling relationship between economic output and carbon emission in the Chinese construction industry. Environ. Impact Assess. Rev..

[6] Sunindijo R.Y., Zou P.X.W. (2012). Political Skill for Developing Construction Safety Climate. J. Constr. Eng. Manag..

[7] National Bureau of Statistics of China (2018). Statistical Communiqué of the People’s Republic of China on the 2017 National Economic and Social Development. http://www.stats.gov.cn/english/PressRelease/201802/t20180228_1585666.html.

[8] China Association of Building Energy Efficiency (2017). Research Report on Building Energy Consumption in China (2017). https://www.sohu.com/a/208615242_99960447.

[9] Chang R.D., Soebarto V., Zhao Z.Y., Zillante G. (2016). Facilitating the transition to sustainable construction: China’s policies. J. Clean. Prod..

[10] Chang R.D., Zuo J., Zhao Z.Y., Soebarto V., Lu Y., Zillante G., Gan X.L. (2018). Sustainability attitude and performance of construction enterprises: A China study. J. Clean. Prod..

[11] Gan X., Zuo J., Ye K., Skitmore M., Xiong B. (2015). Why sustainable construction? Why not? An owner’s perspective. Habitat Int..

[12] Tan Y., Shen L., Yao H. (2011). Sustainable construction practice and contractors’ competitiveness: A preliminary study. Habitat Int..

[13] O’Connor M., Spangenberg J.H. (2008). A methodology for CSR reporting: Assuring a representative diversity of indicators across stakeholders, scales, sites and performance issues. J. Clean. Prod..

[14] Lin X., Ho C.M., Shen G.Q. (2017). Research on corporate social responsibility in the construction context: A critical review and future directions. Int. J. Constr. Manag..

[15] International Organization for Standardization (2010). ISO 26000: Guidance on Social Responsibility. https://www.iso.org/obp/ui/#iso:std:iso:26000:ed-1:v1:en.

[16] Schultz F., Castelló I., Morsing M. (2013). The Construction of Corporate Social Responsibility in Network; Societies: A Communication View. J. Bus. Ethics.

[17] Zhao Z.Y., Zhao X.J., Zuo J., Zillante G. (2016). Corporate social responsibility for construction contractors: A China study. J. Eng. Des. Technol..

[18] Jiang W., Wong J.K. (2016). Key activity areas of corporate social responsibility (CSR) in the construction industry: A study of China. J. Clean. Prod..

[19] Aupperle K.E., Carroll A.B., Hatfield J.D. (1985). An Empirical Examination of the Relationship between Corporate Social Responsibility and Profitability. Acad. Manag. J..

[20] Preston L.E., O’Bannon D.P. (1997). The Corporate Social-Financial Performance Relationship: A Typology and Analysis. Bus. Soc..

[21] Lu W., Ye M., Chau K., Flanagan R. (2018). The paradoxical nexus between corporate social responsibility and sustainable financial performance: Evidence from the international construction business. Corp. Soc. Responsib. Environ. Manag..

[22] Snell R.S. (2000). Studying moral ethos using an adapted Kohlbergian model. Organ. Stud..

[23] Chambers R.G., Serra T. (2018). The social dimension of firm performance: A data envelopment approach. Empir. Econ..

[24] Belu C. (2009). Ranking corporations based on sustainable and socially responsible practices. A data envelopment analysis (DEA) approach. Sustain. Dev..

[25] Aldamak A.M., Zolfaghari S. (2017). Review of efficiency ranking methods in data envelopment analysis. Measure.

[26] Mardani A., Zavadskas E.K., Streimikiene D., Jusoh A., Khoshnoudi M. (2016). A comprehensive review of data envelopment analysis (DEA) approach in energy efficiency. Renew. Sustain. Energy Rev..

[27] Moon H., Min D. (2017). Assessing energy efficiency and the related policy implications for energy-intensive firms in Korea: DEA approach. Energy.

[28] Witte K.D., López-Torres L. (2017). Efficiency in education: A review of literature and a way forward. J. Oper. Res. Soc..

[29] Cheng H.T., Chang H.S. (2018). A Spatial DEA-Based Framework for Analyzing the Effectiveness of Disaster Risk Reduction Policy Implementation: A Case Study of Earthquake-Oriented Urban Renewal Policy in Yongkang, Taiwan. Sustainability.

[30] Hu J.L., Li Y., Tung H.J. (2017). Operational efficiency of ASEAN airlines: Based on DEA and bootstrapping approaches. Manag. Decis..

[31] Liu Q., Wang S., Zhang W., Li J., Zhao Y., Li W. (2017). China’s municipal public infrastructure: Estimating construction levels and investment efficiency using the entropy method and a DEA model. Habitat Int..

[32] Deilmann C., Hennersdorf J., Lehmann I., Reißmann D. (2018). Data envelopment analysis of urban efficiency—Interpretative methods to make DEA a heuristic tool. Ecol. Indic..

[33] Chen C.M., Delmas M. (2011). Measuring corporate social performance: An efficiency perspective. Prod. Oper. Manag..

[34] Zhou H., Yang Y., Chen Y., Zhu J. (2018). Data envelopment analysis application in sustainability: The origins, development and future directions. Eur. J. Oper. Res..

[35] Cai W., Liu F., Xie J., Liu P., Tuo J. (2017). A tool for assessing the energy demand and efficiency of machining systems: Energy benchmarking. Energy.

[36] Yang F.C. (2017). Integrating corporate social responsibility and profitability into best practice selection: The case of large Taiwanese firms. Qual. Quant..

[37] Liu X., Liu J. (2016). Measurement of Low Carbon Economy Efficiency with a Three-Stage Data Envelopment Analysis: A Comparison of the Largest Twenty CO_2_, Emitting Countries. Int. J. Environ. Res. Public Health.

[38] Fried H.O., Lovell C.A.K., Schmidt S.S., Yaisawarng S. (2002). Accounting for Environmental Effects and Statistical Noise in Data Envelopment Analysis. J. Prod. Anal..

[39] Banker R.D., Charnes A., Cooper W.W. (1984). Some Models for Estimating Technical and Scale Inefficiencies in Data Envelopment Analysis. Manag. Sci..

[40] Timmer C.P. (1971). Using a Probabilistic Frontier Production Function to Measure Technical Efficiency. J. Polit. Econ..

[41] Fried H.O., Schmidt S.S., Yaisawarng S. (1999). Incorporating the Operating Environment into a Nonparametric Measure of Technical Efficiency. J. Prod. Anal..

[42] Cui Q., Li Y. (2014). The evaluation of transportation energy efficiency: An application of three-stage virtual frontier DEA. Transp. Res. D.

[43] Shyu J., Chiang T. (2012). Measuring the true managerial efficiency of bank branches in Taiwan: A three-stage DEA analysis. Expert Syst. Appl..

[44] Hsu F.M., Hsueh C.C. (2009). Measuring relative efficiency of government-sponsored R&D projects: A three-stage approach. Eval. Prog. Plan..

[45] Li K., Lin B. (2016). Impact of energy conservation policies on the green productivity in China’s manufacturing sector: Evidence from a three-stage DEA model. Appl. Energy.

[46] Chen Y., Liu B., Shen Y., Wang X. (2016). The energy efficiency of China’s regional construction industry based on the three-stage DEA model and the DEA-DA model. KSCE J. Civ. Eng..

[47] Seiford L.M., Zhu J. (2002). Modeling undesirable factors in efficiency evaluation. Eur. J. Oper. Res..

[48] Aigner D., Lovell C.K., Schmidt P. (1977). Formulation and estimation of stochastic frontier production function models. J. Econom..

[49] Meeusen W., van Den Broeck J. (1977). Efficiency estimation from Cobb-Douglas production functions with composed error. Int. Econ. Rev..

[50] Kumbhakar S.C., Lovell C.K. (2000). Stochastic Frontier Analysis.

[51] China Securities Regulatory Commission (2018). Industry Classification Results of Listed Companies in the 4 Quarter of 2017. http://www.csrc.gov.cn/pub/newsite/scb/ssgshyfljg/201801/t20180119_332958.html.

[52] (2017). Construction Times; Engineering News-Record. “China’s Top 80 Contractors” List. http://www.jzsbs.com/index.php/Home/Index/detail?id=7266.

[53] Buchholtz A.K., Amason A.C., Rutherford M.A. (1999). Beyond resources: The mediating effect of top management discretion and values on corporate philanthropy. Bus. Soc..

[54] China International Contractors Association (2012). Guide on Social Responsibility for Chinese International Contractors. http://images.mofcom.gov.cn/hzs/accessory/201209/1348819602840pdf.

[55] Carroll A.B. (1979). A Three-Dimensional Conceptual Model of Corporate Performance. Acad. Manag. Rev..

[56] Carroll A.B. (1991). The pyramid of corporate social responsibility: Toward the moral management of organizational stakeholders. Bus. Horiz..

[57] Achua J.K. (2008). Corporate social responsibility in Nigerian banking system. Soc. Bus. Rev..

[58] Friedman M. (1970). The Social Responsibility of Business Is to Increase Its Profits. N. Y. Times Mag..

[59] Wan W.J. (2010). Defining corporate social responsibility. J. Public Aff..

[60] The Enterprise Observer (2017). Report of 2017 Top 500 Chinese Charity Enterprise. http://www.cneo.com.cn/article-48517-1.html.

[61] Liu B., Wang X., Chen C., Ma Z. (2014). Research into the dynamic development trend of the competitiveness of China’s regional construction industry. KSCE J. Civ. Eng..

[62] Li J., Chiang Y.H., Choi T.N., Man K.F. (2013). Determinants of efficiency of contractors in Hong Kong and China: Panel data model analysis. J. Constr. Eng. Manag..

[63] EdumFotwe F., Price A., Thorpe A. (2010). A review of financial ratio tools for predicting contractor insolvency. Constr. Manag. Econ..

[64] Pilateris P., Mccabe B. (2003). Contractor financial evaluation model (CFEM). Rev. Can. Genie Civ..

[65] Chen H.L. (2010). Model for Predicting Financial Performance of Development and Construction Corporations. J. Constr. Eng. Manag..

[66] Elyamany A., Basha I., Zayed T. (2007). Performance Evaluating Model for Construction Companies: Egyptian Case Study. J. Constr. Eng. Manag..

[67] Balatbat M.C.A., Lin C.I., Carmichael D.G. (2010). Comparative performance of publicly listed construction companies: Australian evidence. Constr. Manag. Econ..

[68] Severson G.D., Russell J.S., Jaselskis E.J. (1994). Predicting Contract Surety Bond Claims Using Contractor Financial Data. J. Constr. Eng. Manag..

[69] Barney J. (1991). Firm Resources and Sustained Competitive Advantage. J. Manag..

[70] Dyer L., Reeves T. (1995). Human resource strategies and firm performance: What do we know and where do we need to go?. Int. J. Hum. Resour. Manag..

[71] Lopez-Cabrales A., Valle R., Herrero I. (2010). The contribution of core employees to organizational capabilities and efficiency. Hum. Resour. Manag..

[72] Goodrum P.M., Haas C.T. (2004). Long-Term Impact of Equipment Technology on Labor Productivity in the U.S. Construction Industry at the Activity Level. J. Constr. Eng. Manag..

[73] Wu C.L., Fang D.P., Liao P.C., Xue J.W., Li Y., Wang T. (2015). Perception of corporate social responsibility: The case of Chinese international contractors. J. Clean. Prod..

[74] Xiong B., Lu W., Skitmore M., Chau K., Ye M. (2016). Virtuous nexus between corporate social performance and financial performance: A study of construction enterprises in China. J. Clean. Prod..

[75] Zhao Z.Y., Zhao X.J., Davidson K., Zuo J. (2012). A corporate social responsibility indicator system for construction enterprises. J. Clean. Prod..

[76] Hexun The Introduction of Hexun. http://corp.hexun.com/default/index.html.

[77] Li D., Xin L., Chen X., Ren S. (2017). Corporate social responsibility, media attention and firm value: Empirical research on Chinese manufacturing firms. Qual. Quant..

[78] Pan X., Sha J., Zhang H., Ke W. (2014). Relationship between corporate social responsibility and financial performance in the mineral Industry: Evidence from Chinese mineral firms. Sustainability.

[79] Matten D., Moon J. (2008). “Implicit” and “explicit” CSR: A conceptual framework for a comparative understanding of corporate social responsibility. Acad. Manag. Rev..

[80] Wu J. (2014). The antecedents of corporate social and environmental irresponsibility. Corp. Soc. Responsib. Environ. Manag..

[81] Wang X., Fan G., Yu J. (2017). Markefizafion Index of China’s Provinces: NERI Report 2016.

[82] Fan G., Wang X., Zhu H. (2001). NERI Index of Marketization of China’s Provinces.

[83] Muratova Y., Arnoldi J., Chen X., Scholderer J. (2018). Political rotations and cross-province firm acquisitions in China. Asian Bus. Manag..

[84] Li J., Li Z. (2018). Understanding the role of economic transition in enlarging energy price elasticity. Econ. Transit..

[85] Huang D., Liu L. (2014). Micro-determinants of vertical integration: Evidence from China. Asia Pac. J. Manag..

[86] Wang L.H., Dong Z.Q., Huang W.T., Li F.Q. An Empirical Study of the Economic Institution Transition and the Efficiency of Chinese Economic Growth. Proceedings of the International Conference on Management Science and Engineering.

[87] Liu B., Chen Y., Wang R., Shen Y., Shen Q. (2016). Different interaction mechanisms of market structure in the construction industry TFP from the spatial perspective: A case study in China. KSCE J. Civ. Eng..

[88] Kyriacou A.P., Muinelo-Gallo L., Roca-Sagalés O. (2015). Construction corrupts: Empirical evidence from a panel of 42 countries. Public Choice.

[89] Liu W., Wei Q., Huang S.-Q., Tsai S.-B. (2017). doing Good Again? A Multilevel Institutional Perspective on Corporate Environmental Responsibility and Philanthropic Strategy. Int. J. Environ. Res. Public Health.

[90] Li S., Song X., Wu H. (2015). Political Connection, Ownership Structure, and Corporate Philanthropy in China: A Strategic-Political Perspective. J. Bus. Ethics.

[91] Tian Z., Wang R., Yang W. (2011). Consumer responses to corporate social responsibility (CSR) in China. J. Bus. Ethics.

[92] Campbell J.L. (2007). Why Would Corporations Behave in Socially Responsible Ways? An Institutional Theory of Corporate Social Responsibility. Acad. Manag. J..

[93] Ma H., Zeng S., Shen G.Q., Lin H., Chen H. (2016). International diversification and corporate social responsibility. Manag. Decis..

[94] Shen L., Shuai C., Jiao L., Tan Y., Song X. (2017). Dynamic sustainability performance during urbanization process between BRICS countries. Habitat Int..

